# Molecular characterization and elucidation of the function of *Hap38 MAPK* in the response of *Helicoverpa armigera* (Hübner) to UV-A stress

**DOI:** 10.1038/s41598-022-23363-x

**Published:** 2022-11-02

**Authors:** Mengshuang Yao, Xiaofei Liu, Jianyu Meng, Changli Yang, Changyu Zhang

**Affiliations:** 1 Institute of Entomology, Guizhou University, Provincial Key Laboratory for Agricultural Pest Management of Mountainous Regions, Guiyang, 550025 People’s Republic of China; 2 Department of Agriculture and Rural Development, Kaiyang, 550300 Guiyang People’s Republic of China; 3 Guizhou Tobacco Science Research Institute, Guiyang, 550081 People’s Republic of China

**Keywords:** Molecular biology, Environmental sciences

## Abstract

The cotton bollworm *Helicoverpa armigera* (Hübner) (Lepidoptera: Noctuidae), an important pest of cotton, is detrimental to cotton production. Light from UV-A ultraviolet lamps is regarded as a form of environmental stress for insects. In order to investigate the response of *H. armigera* exposed to UV-A, we explored *Hap38 MAPK* expression and functions. We hope that the findings of this study will lay the foundation for future investigations into the insect’s phototaxis mechanism. A *p38 MAPK* was cloned and named *Hap38 MAPK*. A phylogenetic tree showed that *Hap38 MAPK* was highly conserved. The gene was highly expressed in the thorax and females. Under UV-A stress, the expression of the gene decreased significantly. After silencing *Hap38 MAPK*, the activity of the antioxidant enzymes SOD, POD, CAT, and GR decreased. This study suggested that *Hap38 MAPK* responds to UV-A irradiation and plays critical roles in the defense response to environmental stresses.

## Introduction

The cotton bollworm *Helicoverpa armigera *(*Hübner*) (*Lepidoptera: Noctuidae*), is an omnivorous pest of food crops distributed worldwide, which causes enormous yield losses annually^1^. It has a wide host range and exhibits rapid reproduction, high adaptability, and high pesticide resistance^2^. Because *H. armigera* exhibits typical phototaxis^3^, research into this pest has been focused upon the photoreactive and phototactic characteristics and microscopic structure of its compound eyes, as well as the effects of the compound eye state on phototactic behaviors^4–6^. Previous studies have demonstrated that the visual physiology and behavioral characteristics of *H. armigera* are highly sensitive to ultraviolet (UV) light. In recent years, UV-A irradiation has received increasing attention as an important stress factor, causing a series of direct or indirect adverse effects, such as oxidative stress, photoreceptor damage, and apoptosis^7^, in almost all organisms, including insects. After UV-A irradiation, the metabolism of juvenile hormones of *H. armigera* was affected, oviposition was increased, and the life span was shortened^8–10^.

As a phenomenon, phototaxis of insects that make UV-A ultraviolet insect trap lamp are widely used. As an environmental stress factor, UV-A causes excessive reactive oxygen species (ROS) in insects, resulting in varying degrees of oxidative damage to some biological macromolecules^11,12^. These damages will cause a series of biological effects on insects, such as toxin production, mutations, and changes in signal transduction pathways^13^. MAPK was upregulated following UV light irradiation in insects. Insects respond to UV stress by changing enzyme system activity, juvenile hormone metabolism, and protein expression^9,14^. Insects can apparently perceive signals using sensors and transmit them to the cellular machinery via signal transduction to regulate gene expression. This study mainly elucidates the function of *Hap38 MAPK* in response to UVA stress. Mitogen-activated protein kinases (MAPKs) are a large group of protein kinases that perform a wide variety of roles in cellular signal transduction pathways^15^. There are at least four distinct MAPK signaling modules, which mediate extracellular signals into the nucleus, to activate responsive genes. These include p38 mitogen-activated protein kinase (p38 MAPK), c-Jun N-terminal kinase (JNK), and extracellular signal-regulated kinase (ERK)^16,17^. Of the above three proteins, p38 MAPK is easily induced by a variety of environmental stresses, such as temperature change, UV radiation, and other external stimuli^18^. An important member of this family, p38 MAPK, plays a key role in inflammation and stress response, and participates in the regulation of cell survival, differentiation, and apoptosis. The activation of p38 MAPK protein is stimulated by various stresses, such as those induced by extracellular inflammatory factors, UV radiation, and cytotoxic substances^19,20^. Activated p38 MAPK regulates cell function by regulating the expression of downstream genes encoding various enzymes and transcription factors^21^. There have been many studies into the stress-induced apoptosis of immune cells, depending on p38 MAPK signaling pathways. For instance, the p38 MAPK signaling pathway is involved in the regulation of heat shock protein 70 (Hsp70), and protects the red blood cells of *Sparus aurata* exposed to heat stress^22^. UV-A can selectively activate p38 MAPK in C6 glioma cells of the rat, which in turn enables p38 MAPK to protect the cells from radiation^23^. In insects, a novel peptide from *Vespa ducalis* induces apoptosis in osteosarcoma cells by activating p38 MAPK^24^. This paralytic peptide has been shown to enhance p38 MAPK phosphorylation following induction, initiating immune responses in embryonic cells in *Bombyx mori*^25^. In particular, it was shown that *Drosophila* mutants lacking D-MEKK1 were hypersensitive to environmental stresses, including elevated temperature and increased osmolarity^26^. Therefore, the p38 MAPK pathway is critical for the response to environmental stresses in insects.

ROS normally exist in cells in a state of equilibrium between production and elimination. Environment stress causes this balance to be disturbed^27^. To scavenge excess ROS and reduce its toxic effects, insects have evolved an antioxidant system whose activity is upregulated under stressful conditions, and is correlated well with enhanced tolerance. The system involves superoxide dismutase (SOD), peroxidase (POD), catalase (CAT), and glutathione reductase (GR)^28^. The first three enzymes act in coordination to maintain the normal physiological activities of insects. The activity levels of SOD, POD, and CAT in *Pieris rapae* treated with deltamethrin were higher than those of normal insects, in order to adapt to external toxicity^29^. GR is the key enzyme of the ascorbic acid glutathione (AsA-GSH) cycle, and can play an indirect antioxidant role^30^. Injection of *Escherichia coli* into *Ostrinia furnacalis* larvae activates GR, leading to the scavenging of excessive ROS, to prevent against their toxic effects^31^. Under drought stress, salt stress, and cold stress, the activities of SOD, POD, and CAT in maize decrease to varying degrees after the activity of MAPK cascade pathway is inhibited, and the response to stress signals becomes slow. The MAPK pathway participates in the response of maize seedlings to low temperature stress^32^. The MAPK pathway and enzymes to remove ROS, therefore appear to be involved in regulating the antioxidant enzyme defense response of organisms to stress.

In this study, a gene encoding p38 MAPK in *H. armigera* was sequenced, characterized, and compared to homologous proteins from other insect species. We also produced tissue- and stage-specific developmental profiles, and investigated the expression levels of *p38 MAPK* under UV-A irradiation. To explore the role of p38 MAPK in the antioxidant system of *H. armigera* under UV-A stress, the activities of SOD, POD, CAT, and GR were investigated in *H. armigera* with silenced *Hap38 MAPK*. The aims of this work were to reveal the role of the *p38 MAPK* gene in the response to environmental stress and the regulation of abiotic stress in antioxidant defenses, to explore the molecular mechanisms of the response of *H. armigera* to environmental stress, and to provide a theoretical framework for formulating scientific control strategies.

## Materials and methods

### Insect rearing and tissue sampling

Larvae of *H. armigera* were collected from cotton plants growing in a suburb of Huaxi, Guiyang, Guizhou, China, and subsequently reared in an insectary for several generations at 27 ± 1 °C, 75 ± 5% relative humidity, and a 14 h light/10 h dark photoperiod (light, 06:00–20:00; dark, 20:00–06:00). The larvae were fed with an artificial diet, as described previously^33^. Within 3 days of the pupae emerging as adults, one male and one female adult were selected from the cages (20 cm × 20 cm × 30 cm), and paired in a plastic container (15 cm height and 9 cm diameter) with 10% honey solution feeding. *H. armigera* samples were collected at nine developmental stages: egg, 1st–6th instar larvae, 1-, 3-, 5-, and 7-day-old pupae, and 3-day-old adults (male and female). A total of 50 eggs and 8–15 individuals at other developmental stages were collected per biological replicate. Nine different tissues were collected from *H. armigera* adults, including the head (without antennae or compound eyes), chest, abdomen, antennae, compound eyes, feet, wings, midgut, and ovary (in females). Experiments were performed in triplicate. All samples were immediately frozen in liquid nitrogen and stored at − 80 °C until needed for RNA extraction. Three biological replicates were prepared for each treatment three times for each process.

### UV-A irradiation

UV-A light (315–400 nm; Nanjing Huaqiang Electronics Co., Ltd., Nanjing, China) was used to irradiate the adults of *H. armigera* at an irradiance of 300 mW/cm^2^. 150 female and 150 male adults were exposed individually to UV-A light 2 h after the start of scotophase, at a temperature of 27 ± 1 °C. Eight adults per treatment were randomly selected at the start of the experiment, at 0 min (control) and subsequently at 30, 60, 90, 120, and 150 min. There were three technical replicates; 144 samples were collected. Samples were immediately frozen in liquid nitrogen and stored at − 80 °C for subsequent RNA extraction.

### RNA extraction and cDNA synthesis

Total RNA was extracted from 3-day-old *H. armigera* adults using TRIzol reagent (Invitrogen, Carlsbad, CA, USA), according to the manufacturer’s instructions. To remove traces of contaminating genomic DNA, total RNA was treated with DNase I (Takara Bio Inc., Shiga, Japan). To evaluate the purity of the total RNA, the 260/280 ratio was measured using a NanoDrop 2000 (Thermo Fisher Scientific, Waltham, MA, USA). The integrity of the total RNA was examined using gel electrophoresis with a 1% agarose gel. The first-strand cDNA was synthesized immediately from 1 μg of total RNA using RevertAid First-Strand cDNA Synthesis Kit (Thermo Fisher), according to the manufacturer’s instructions, and stored at − 20 °C until needed for further analysis.

### Cloning and sequence analysis of the Hap38 MAPK gene

The *Hap38 MAPK* gene was amplified from the cDNA template by reverse transcription PCR (RT-PCR). The primers used for the RT-PCR (Table S1) were designed using Primer Premier 6.0 (Premier Biosoft International, Palo Alto, CA, USA) to amplify the conserved regions of the *p38 MAPK* genes in *H. armigera*. RT-PCR was carried out using Taq polymerase (Sangon Biotech, Shanghai, China) under the following conditions: initial denaturation at 95 °C for 3 min, followed by 35 cycles of denaturation at 95 °C for 30 s, annealing at 54 °C for 30 s, elongation at 72 °C for 1 min, and a final elongation at 72 °C for 10 min. To amplify the 5′ and 3′ ends of the *Hap38 MAPK* gene, 5′ rapid amplification of cDNA ends (5′ RACE) and 3′ RACE PCRs were carried out, respectively, using SMARTer^®^ RACE 5′/3′ Kits (Clontech, Mountain View, CA, USA), and sequence-specific primers designed using Primer Premier 6.0. The RACE PCRs were performed under the following conditions: 25 cycles of denaturation at 94 °C for 30 s, annealing at 55–65 °C (depending on the primer pair) for 30 s, and extension at 72 °C for 3 min. The PCR products were separated on 1% agarose gel, and the expected bands were gel-purified and cloned. The cloned fragments were then sent to Sangon Biotech for sequencing.

The open reading frame of the gene was identified using ORF finder (http://www.ncbi.nlm.nih.gov/orffinder/), and the amino acid sequence of the encoded protein was determined. The NCBI BLAST (https://blast.ncbi.nlm.nih.gov/Blast.cgi^34^) database was used to analyze the homology of the *Hap38 MAPK* gene with its orthologs in other insects. The molecular weight and isoelectric point (pI) of the p38 MAPK proteins were predicted using the ExPaSy proteomics server (http://www.expasy.org). The protein domains of p38 MAPK were predicted using ExPaSy-PROSITE (http://prosite.expasy.org/). O-glycosylation sites were analyzed using NetOGlyc 4.0 Server (http://www.cbs.dtu.dk/services/NetOGlyc/). The N-terminal signal peptide positions were determined using SignalP 4.1 Server (http://www.cbs.dtu.dk/services/SignalP/). The family signature sequence of Hap38 MAPK was analyzed using ExPASy ScanProsite (http://prosite.expasy.org/scanprosite/). Phylogenetic analysis of p38 MAPK amino acid sequences was performed using the neighbor-joining method and a bootstrap test with 1000 replicates in MEGA6.

### Effect of dsRNA treatment on *Hap38 MAPK*

dsRNA synthesis primers were designed based in the cDNA sequence of *Hap38 MAPK* obtained by cloning, and T7 transcription promoters were added to both ends of the target area. Using the recovered high concentration cDNA as a template, dsRNA was prepared according to the instructions from the MEGAscript^®^ kit (Thermo Fisher Scientific). The dsRNA was purified and tested, and stored at − 80 °C.

Six-day pupae were injected with 6 μL PBS solution (control groups) and 1 μg *Hap38 MAPK*-dsRNA (experimental groups); overall, 60 pupae were tested. After they grew to 3-day-old adults, the tissue samples were collected and the expression levels of *Hap38 MAPK* were measured. The adults were irradiated with a UV-A lamp, at an intensity of 300 μW/cm^2^ for 60 min, under temperature and humidity consistent with the normal feeding conditions. After treatment, the samples were frozen in liquid nitrogen. Each treatment had three biological repeats, and each biological repeat contained eight adults.

Antioxidase activity was determined using the SOD, POD, CAT, and GR kits produced by Suzhou Keming Biotechnology Co., Ltd. (Suzhou, China). The *H. armigera* adults from the different treatment groups were mixed with 150 µL of lysate per 3 mg adult, crushed in a high-speed ball mill for 2 min, centrifuged at 4 °C and centrifuged at 1500 r/min for 10 min. The supernatant was removed, and the protein concentration was measured using the aforementioned quantitative detection kits. The concentration of extracted protein in each group was balanced according to the instruction manual for the SOD, POD, CAT, and GR enzyme activity test kits. The reaction system was added to a 96-well enzyme plate, incubated at 37 °C for color development, the A470 value detected using an enzyme marker, and the protein content calculated.

### Quantitative real-time PCR (qRT-PCR) analysis

The extraction of total RNA and synthesis of cDNA from the different tissue types and developmental stages was performed as described above. The relative expression levels of the *Hap38 MAPK* gene were measured using qRT-PCR with FastStart Essential DNA Green Master Mix (Roche, Indianapolis, IN, USA) on a CFX96™ Real-Time Quantitative PCR system (Bio-Rad, Hercules, CA, USA). The primers used for qRT-PCR are listed in Table 1. qRT-PCR was conducted in a 20 μL reaction volume containing 1 μL of cDNA template, 1 μL of each forward and reverse primer (10 μM), 7 μL of diethyl pyrocarbonate-treated H_2_O, and 10 μL of FastStart Essential DNA Green Master Mix. The cycling parameters used for qRT-PCR were as follows: 95 °C for 10 min, followed by 40 cycles at 95 °C for 30 s and 60 °C for 30 s. Melting curves were generated at 65–95 °C. β-Actin (GenBank accession no.: HM629441.1) was used as an internal control. To verify reproducibility, each qRT-PCR was performed using three technical replicates and three biological replicates. Relative gene expression was calculated using the 2^−ΔΔCT^ method^35^. The expression levels of the genes in the adult males, head (antennae and compound eyes were removed), and at 0 min, were normalized.

**Table 1:** Names and accession numbers of genes used in the phylogenetic tree analysis.

Gene name	Species	Genebank number
Hap38 MAPK	*Helicoverpa armigera*	MK185422
Slp38 MAPK	*Spodoptera litura*	XM_022966475.1
Tnp38 MAPK	*Trichoplusia ni*	XM_026883270.1
Pxp38 MAPK	*Vanessa tameamea*	XM_026642141.1
Ppp38 MAPK	*Galleria mellonella*	XM_026902837.1
Bap38 MAPK	*Amyelois transitella*	XM_013341701.1
Vtp38 MAPK	*Athalia rosae*	XM_012407911.2
Plxp38 MAPK	*Zootermopsis nevadensis*	XM_022086633.1
Atp38 MAPK	*Papilio polytes*	XM_013277843.1
Gmp38 MAPK	*Papilio xuthus*	XM_013321935.1
Mpp38 MAPK	*Bicyclus anynana*	XM_024080016.1
Sip38 MAPK	*Plutella xylostella*	XM_011563257.1
Ccp38 MAPK	*Neodiprion lecontei*	XM_015653904.1
Vep38 MAPK	*Harpegnathos saltator*	XM_011139608.3
Hsp38 MAPK	*Dinoponera quadriceps*	XM_014632703.1
Dqp38 MAPK	*Vollenhovia emeryi*	XM_012005168.1
Arp38 MAPK	*Frankliniella occidentalis*	XM_026417006.1
Nlp38 MAPK	*Monomorium pharaonic*	XM_012677991.1
Nilp38 MAPK	*Cyphomyrmex costatus*	XM_018544516.1
Znp38 MAPK	*Solenopsis invicta*	XM_026134947.1
Fop38 MAPK	*Nilaparvata lugens*	XM_022338737.1
Amp38 MAPK	*Ailuropoda melanoleuca*	XM_011218520.1

### Statistical analysis

Differences between treatments were analyzed using one-way analysis of variance (ANOVA), followed by Duncan’s multiple comparison tests. All analyses were performed using SPSS version 21.0 (SPSS, Chicago, IL, USA). Data are presented as mean ± standard error (SE) of three biological replicates. *P* < 0.05 was considered a statistically significant difference, which is clearly illustrated using different letters or * in figure note.

## Results

### Cloning and sequencing of Hap38 MAPK

**Figure 1:**
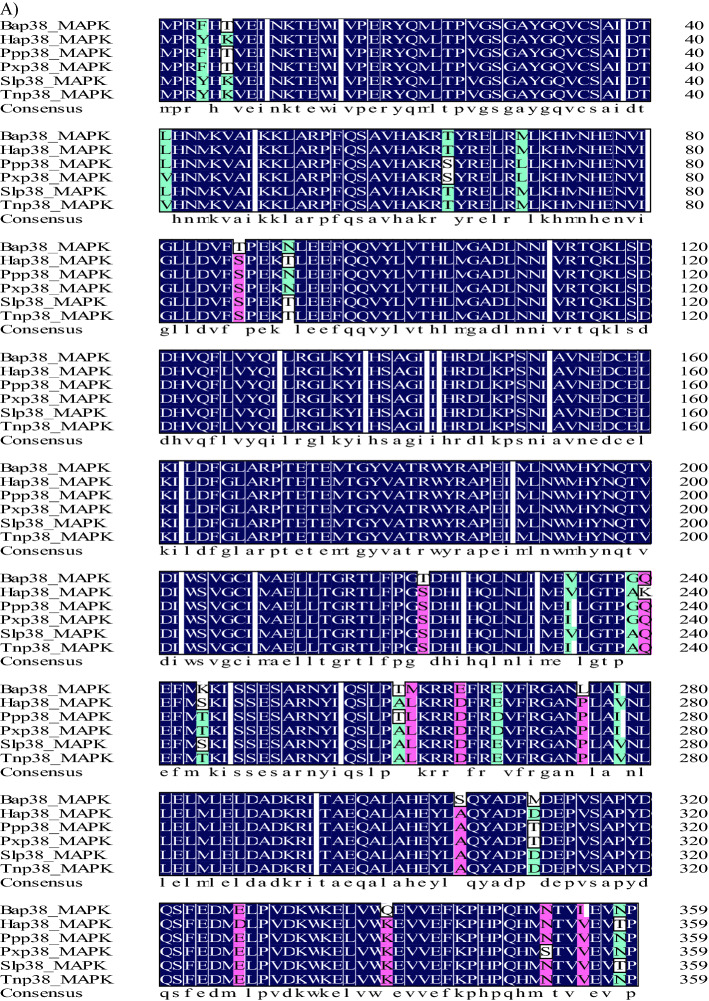

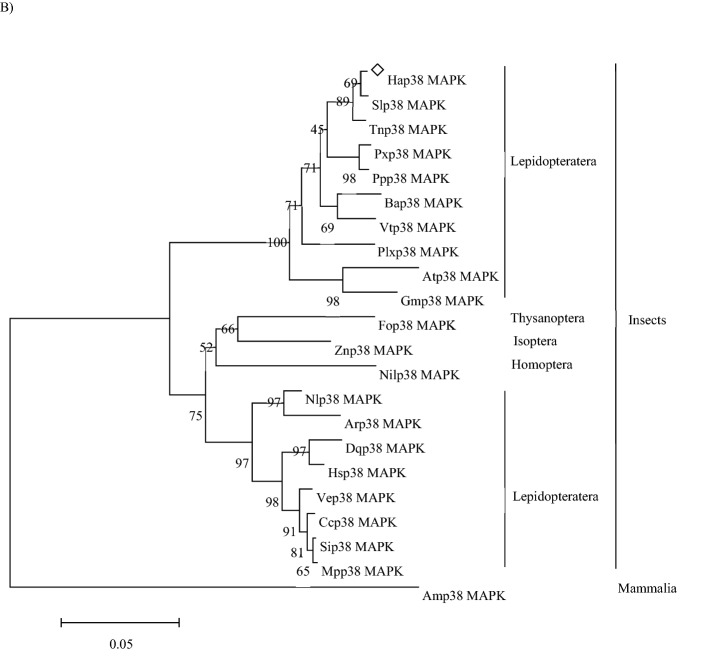
Sequence characterization of p38 MAPK from various species. (**A**) Multiple alignments of the amino acid sequences of Hap38 MAPK with homologs from other insect species. Black represents 100% identity, red represents ≥ 75% identity, green represents ≥ 50% identity, and white represents < 50% identity. Hap38 MAPK (*Helicoverpa armigera*, MK185422), Slp38 MAPK (Spodoptera *litura*, XP_022822243.1), Tnp38 MAPK (*Trichoplusia ni*, XP_026739071.1), Ppp38 MAPK (*Papilio polytes*, XP_013133297.1), Pxp38 MAPK (*Papilio xuthus*, XP_013177389.1), and Bap38 MAPK (*Bicyclus anynana*, XP_023935784.1). (**B**) Phylogenetic tree of p38 MAPKs from *H. armigera* (Ha) and other species. The tree was constructed from a multiple alignment using the MEGA6.0 software, and generated with 1000 bootstrap trials using the neighbor-joining method. The numbers show the bootstrap confidence values obtained for each node after 1000 repetitions.

A *p38 MAPK* gene was cloned from *H. armigera* and named *Hap38 MAPK* (GenBank accession no.: MK185422). This gene contains an open reading frame of 1080 bp, encoding 360 amino acids. The putative protein is 41.51 kDa and has a theoretical pH(I) of 5.93. Amino acid sequence analysis using SignalP revealed that the Hap38 MAPK protein contained no signal peptide or transmembrane region, but NetOGlyc identified three O-glycosylation sites and ExPASy ScanProsite identified two domains: a substrate-binding site at positions 25–50, and a threonine-proline-tyrosine motif at 55–158 (Fig. 1A). The sequences of 22 p38 MAPK sequences, including Hap38 MAPK and 21 other p38 MAPK protein sequences from GenBank (Table 1), were aligned and used for homology comparison and the construction of a phylogenetic tree (Fig. 1B). The phylogenetic tree and homology comparison showed that the p38 MAPK proteins were closely related in different Lepidopteran insects. The Hap38 MAPK protein was most closely related to Slitp38 MAPK (99.44%), Tnip38 MAPK (98.33%), Ppolp38 MAPK (95.83%), and Pxutp38 MAPK (95.56%).

### Developmental expression profiles of tissue-specific Hap38 MAPK

**Figure 2:**
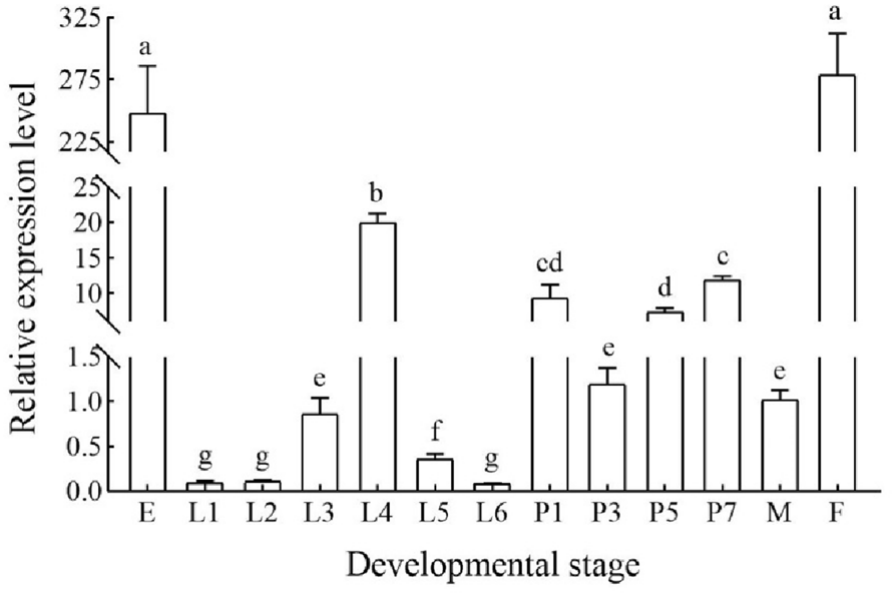
Relative expression levels of Hap38 MAPK in different developmental stages of *H. armigera*. E (Egg), L1–L6 (1st–6th instar larva), P1, P3, P5, P7 (1-, 3-, 5-, and 7-day-old pupa), F (3-day-old adult females) and M (3-day-old adult males). The expression levels of the genes in the adult males are normalized. Data in the figure are expressed as mean ± SE. Different letters above bars indicates significant difference. The values with same superscript letters in the same line are of no significant difference (*P* > 0.05), those with different letters are of significant or extreme difference (*P* < 0.05, ANOVA).

**Figure 3:**
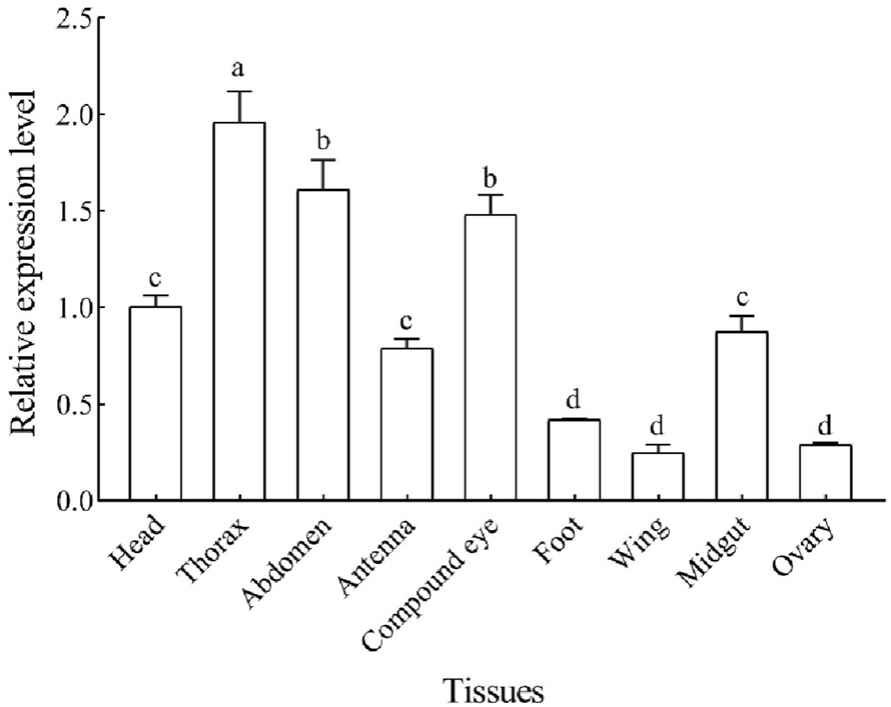
Relative expression levels of *Hap38 MAPK* in different adult tissues of *H. armigera*. The expression levels of genes in the head were normalized. Data in the figure are expressed as mean ± SE. Different letters above bars indicates significant difference (*P* < 0.05, ANOVA).

The expression profiles of *Hap38 MAPK* at different developmental stages were quantified. The expression of *Hap38 MAPK* was significantly higher in the egg and 3-day-old adult females than in other stages (*p* < 0.05) (Fig. 2). There were significant differences in the expression of *Hap38 MAPK* in different tissues; specifically, that in the thorax was the highest, followed by those in the abdomen and compound eye (Fig. 3).

### Expression of Hap38 MAPK after exposure to UV-A

**Figure 4:**
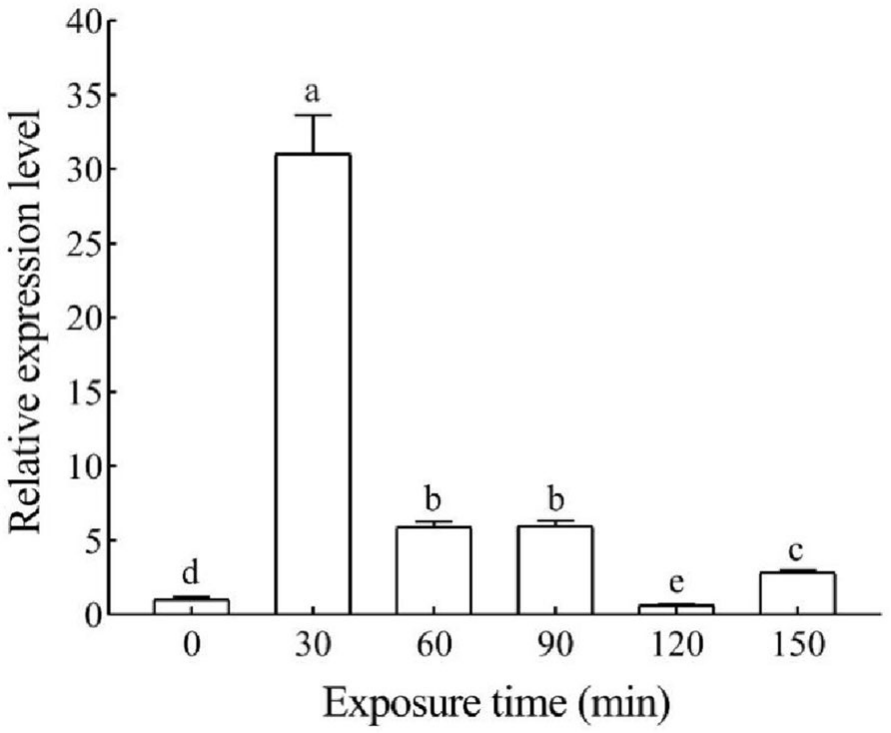
Relative expression levels of *Hap38 MAPK* in different UV-A treatments of *H. armigera*. The expression levels of genes at 0 min are normalized. Data in the figure are expressed as mean ± SE. Different letters above bars indicate significant differences (*P* < 0.05, ANOVA).

UV-A treatment induced the expression of *Hap38 MAPK* in adults; however, significant differences were detected in the expression of *Hap38 MAPK* between samples exposed to UV-A for different time periods (*p* < 0.05) (Fig. 4). The expression of *Hap38 MAPK* first increased in response to UV-A, reaching a peak at 30 min, and then decreased with the increase in treatment time. The expression of *Hap38 MAPK* was significantly higher at 30, 60, and 90 min of UV-A treatment than in the control. However, at 120 min, the expression level of *Hap38 MAPK* returned to the level of the control.

### dsHap38 MAPK-RNA inhibited the expression of the *Hap38 MAPK* gene

**Figure 5:**
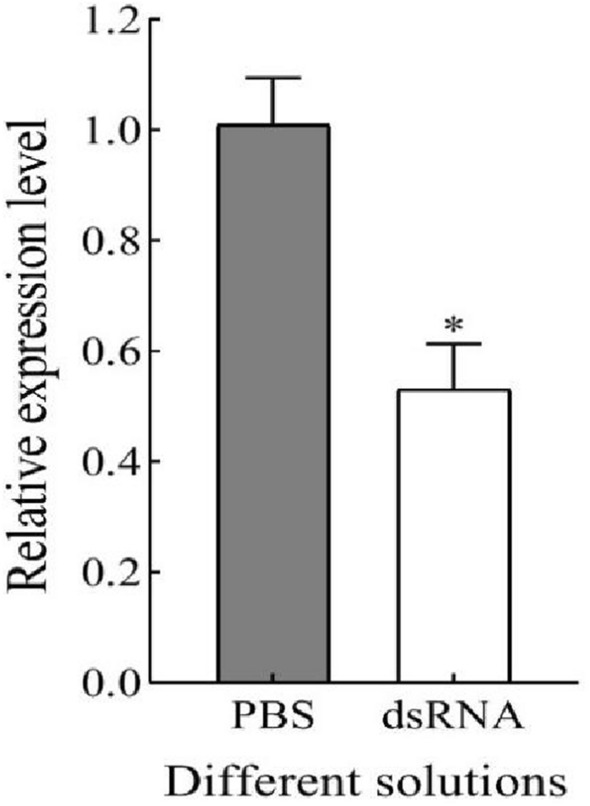
Detection of silencing efficiency. The relative expression levels of *Hap38 MAPK* were detected in adults. The bars represent means ± SE and asterisks on bars indicate significant differences. The sample size was 50 adults (*P < 0.05, ANOVA).

To further explore the role of *Hap38 MAPK* in the regulation of *H. armigera*, the effect of RNAi-based silencing on *Hap38 MAPK* gene expression was observed by injecting *Hap38 MAPK*-dsRNA. Compared with the control group, the expression of the *Hap38 MAPK* gene was significantly decreased in the gene silenced groups (Fig. 5, F = 0.006; df = 4; P = 0.016).

### Effect of *Hap38* MAPK-dsRNA on the activity of antioxidant enzymes under UV-A

**Figure 6:**
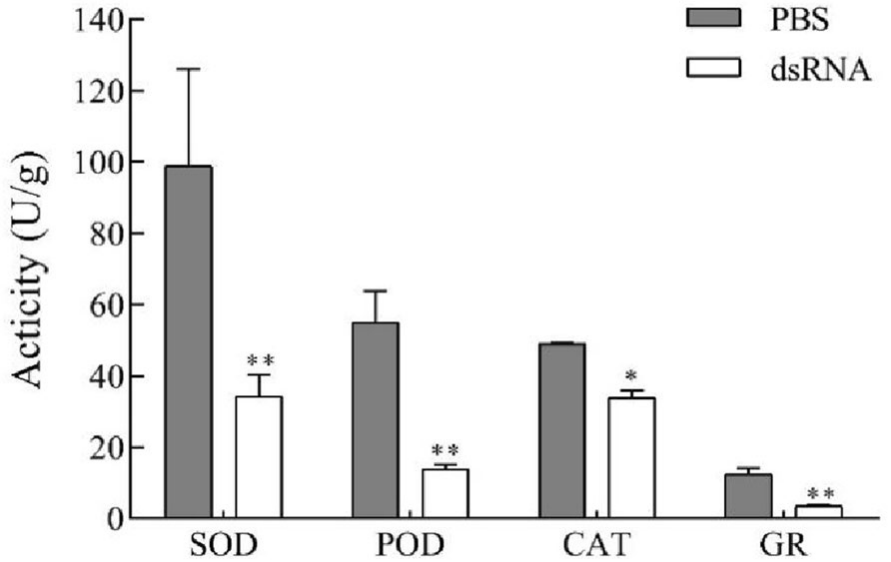
Effects of UV-A radiation on SOD, POD, CAT, and GR activity of *H. armigera* 60 min after injection with PBS or dsRNA. Data in the figure are expressed as means ± SE (*P < 0.05, **P < 0.01, ANOVA).

Under UV-A stress, a series of antioxidant enzymes are secreted to facilitate adaptation to the environment. Our previous study confirmed the hypothesis that UV light irradiation increases the level of oxidative stress in *H. armigera* adults11. As a class of antioxidant enzymes, SOD, POD, CAT, and GR are likely to be transported to their destination via processing involving *Hap38 MAPK*. Under UV-A stress for 60 min, the activities of SOD, POD, CAT, and GR were significantly lower than those of control after the silencing of *Hap38 MAP*K. This observation indicated that p38 MAPK signal transduction was blocked in *H. armigera* injected with *Hap38 MAPK*-dsRNA. ROS increased in the body, and the peroxidation damage to the plasma membrane and membrane lipids by free radicals increased (Fig. 6).

## Discussion

Previous studies on insects have shown that p38 MAPK plays a pivotal role in signal transduction pathways, and is activated by UV light. To explore the biological function, a *p38 MAPK* gene was cloned and named *Hap38 MAPK*, which contains an open reading frame of 1080 bp, encoding 360 amino acids, no signal peptide or transmembrane regions. These findings are consistent with the characteristics of *p38 MAPK* known from other insect species^36,37^. The *p38 MAPK* gene of *H. armigera* had a relatively conservative evolutionary relationship in Lepidoptera and other insects.

To study the expression and physiological functions of *H. armigera*, the expression level of *Hap38 MAPK* was detected. The findings were consistent with the mammalian^38^. The expression level of *Hap38 MAPK* was the highest in the thorax of adults. The activation of the p38 MAPK signal pathway can regulate skeleton^39^. Therefore, the high expression level in the chest may be related to regulating muscle differentiation of *H. armigera*^40,41^. The expression level of *Hap38 MAPK* in females were significantly higher, this difference might be determined by the phosphorylation^42^. It can be inferred that the p38 MAPK signaling pathway plays a critical role in the follicular development of *H. armigera*^43^. *Hap38 MAPK* plays an important regulating role about information transmission and death^44^.

Insects have strong phototaxis, Entomologists have carried out a considerable amount of research into the effects of UV-A on insects. In this study, the expression level of *Hap38 MAPK* was significantly increased when UV-A 30 min. Short-term UV-A irradiation induces stress in *H. armigera* adults, leading to the phosphorylation of Hap38 MAPK and activation of the p38 MAPK pathway, which further leads to the insects to resist UV stress and improve adaptability^45,46^. Apoptosis in *Bombyx mori* cells is regulated by p38 MAPK^47^. The extent of the stress response of *H. armigera* increased with an increase in the duration of UV-A irradiation. There is an apparent balance between p38 MAPK and JNK signaling in response to the stress. The sustained activation of p38 MAPK resulted in the activation of c-JNK and extracellular signal-regulated protein kinase, which in turn inhibited p38 MAPK expression^48,49^. The interaction of pathways resulted in a decrease in expression levels below the control level at UV-A 120 min. Our results confirm the hypothesis that UV light irradiation increases the expression level of *Hap38 MAPK* in *H. armigera* adults.

ROS is normally in a state of dynamic equilibrium in organisms, this balance is disrupted by UV-A^50^. As a part of the defense system, SOD, POD, CAT, and GR can maintain the balance of ROS^51,52^. As an exogenous factor, Meng et al., studied the antioxidant response of *H. armigera* to UV-A stress, UV light irradiation activated MAPK signal transduction and increased the level of oxidative stress in *H. armigera* adults^11^. Meng et al*.*, studied found that exposure to UV light for 30 min resulted in increased total antioxidant capacity, protein carbonyl content and activities of SOD, CAT, POX and GST, and the result confirmed that UV light irradiation increases the level of oxidative stress in *H. armigera* adults. Now we investigated the expression of the *Hap38 MAPK* gene in dsRNA-injected group compared with the control under UV-A. After silencing the gene, *p38MAPK* activity was significantly inhibited, possibly affecting the scavenging of ROS and the improvement of antioxidant capacity. When dsRNA-injected pupae were exposed to UV-A, the activity of SOD, POD, CAT, and GR increased in a short time, indicating that the activity of enzymes was correlated with tolerance to a negative environment^11,53^. We inferred the different antioxidant enzymes of *H. armigera* act in a coordinated manner against the stress. Similar effects have been observed in *Myzus persicae*^54^*.* The p38 MAPK signaling pathway was inhibited, it will affect other pathways^55^. Some drugs have been found to inhibit and improve the level of JNK under stress, as reflected by the levels of SOD and CAT. This process is consistent with our study, which may be related to the enzymes’ regulatory effect on the expression of *p38 MAPK*. The effects of UV exposures on adult longevity and reproduction in *H. armigera* were investigated in our previous research^8^. Exposure to UV-A for longer periods caused a decline in cumulative survival of F_1_ immature stages. These results suggested that the response mechanisms of insect to UV light irradiation stress is complicate.

## Conclusions

In the present study, we investigated the expression patterns of the *Hap38 MAPK* gene in different tissues, at different stages of development, and under UV-A stress. We characterized the function of *Hap38 MAPK*, and found that the injection of dsRNA could significantly reduce the expression of *Hap38 MAPK* and change the activity of antioxidant enzymes. These findings indicate that *Hap38 MAPK* responds to UV-A stress and has a close relationship with biological antioxidant functions.

## Supplementary Information

Supplementary Information 1.

Supplementary Information 2.

Supplementary Information 3.

Supplementary Information 4.

Supplementary Information 5.

Supplementary Information 6.

Supplementary Information 7.

Supplementary Information 8.

Supplementary Information 9.

Supplementary Information 10.

## Data Availability

The datasets generated and/or analysed during the current study are available in the [Genbank] repository, accession number to datasets [https://www.ncbi.nlm.nih.gov/, MK185422, XM_022966475.1, XM_026883270.1, XM_026902837.1, XM_013341701.1, XM_012407911.2, XM_022086633.1, XM_013277843.1, XM_013321935.1, XM_024080016.1, XM_011563257.1, XM_015653904.1, XM_011139608.3, XM_014632703.1, XM_012005168.1, XM_026417006.1, XM_012677991.1, XM_018544516.1, XM_026134947.1, XM_022338737.1, XM_011218520.1].
