# Supplementary material for: Molecular characterization and elucidation of the function of Hap38 MAPK in the response of Helicoverpa armigera (Hübner) to UV-A stress
Source: Sci Rep. 2022 Nov 2;12:18489. doi: 10.1038/s41598-022-23363-x (PMC9630311; doi:10.1038/s41598-022-23363-x)
Supplement: Supplementary file 9 — Supplementary Information 9. [file 41598_2022_23363_MOESM9_ESM.docx]

Table S1 Nucleotide primers used to obtain the full-length cDNA of Hap38 MAPK by degenerate PCR, RACE PCR and qRT-PCR

| Primers | Primers sequences | Use of primer | |
| --- | --- | --- | --- |
| Hap38 MAPK - F  Hap38 MAPK - R | GCAACAGAATGTCGCCATCA  GTCAGTCCATACGCAGAGAATC | | Amplificating of *Hap38 MAPK* gene fragment |
| Hap38 MAPK 3' – GSP  Hap38 MAPK 3'–N GSP | GATTACGCCAAGCTTCTAGGAACTACATTCAGTCTCT  GATTACGCCAAGCTTCGTATAACAGCGGAACAAG | | 3' RACE |
| Hap38 MAPK q-F  Hap38 MAPK q-R | CGCTCACGAATACTTGGCTCAA  GTGTTCATGTGTTGTGGGTGGG | | Target gene amplification by RT-qPCR |
| Actin-F  Actin-R | AATCGTGCGTGACATCAA  AGGAAGGAAGGCTGGAAG | | Reference gene amplificationby RT-qPCR |
